# Does a youth intern programme strengthen HIV service delivery in South Africa? An interrupted time‐series analysis

**DOI:** 10.1002/jia2.26083

**Published:** 2023-04-12

**Authors:** Deanna Tollefson, Sayan Dasgupta, Geoffrey Setswe, Sarah Reeves, Gavin Churchyard, Salome Charalambous, Ann Duerr

**Affiliations:** ^1^ Department of Global Health University of Washington Seattle Washington USA; ^2^ Fred Hutchinson Cancer Research Centre Vaccine and Infectious Disease & Public Health Science Divisions Seattle Washington USA; ^3^ The Aurum Institute Johannesburg South Africa; ^4^ Department of Health Studies University of South Africa Pretoria South Africa; ^5^ Youth Health Africa Johannesburg South Africa; ^6^ School of Public Health University of the Witwatersrand Johannesburg South Africa

**Keywords:** HIV infections/prevention and control, community health workers, health workforce, delivery of healthcare, quasi‐experimental studies

## Abstract

**Introduction:**

Since 2018, Youth Health Africa (YHA) has placed unemployed young adults at health facilities across South Africa in 1‐year non‐clinical internships to support HIV services. While YHA is primarily designed to improve employment prospects for youth, it also strives to strengthen the health system. Hundreds of YHA interns have been placed in programme (e.g. HIV testing and counselling) or administrative (e.g. data and filing) roles, but their impact on HIV service delivery has not been evaluated.

**Methods:**

Using routinely collected data from October 2017 to March 2020, we conducted an interrupted time‐series analysis to explore the impact of YHA on HIV testing, treatment initiation and retention in care. We analysed data from facilities in Gauteng and North West where interns were placed between November 2018 and October 2019. We used linear regression, accounting for facility‐level clustering and time correlation, to compare trends before and after interns were placed for seven HIV service indicators covering HIV testing, treatment initiation and retention in care. Outcomes were measured monthly at each facility. Time was measured as months since the first interns were placed at each facility. We conducted three secondary analyses per indicator, stratified by intern role, number of interns and region.

**Results:**

Based on 207 facilities hosting 604 interns, YHA interns at facilities were associated with significant improvements in monthly trends for numbers of people tested for HIV, newly initiated on treatment and retained in care (i.e. loss to follow‐up, tested for viral load [VL] and virally suppressed). We found no difference in trends for the number of people newly diagnosed with HIV or the number initiating treatment within 14 days of diagnosis. Changes in HIV testing, overall treatment initiation and VL testing/suppression were most pronounced where there were programme interns and a higher number of interns; change in loss to follow‐up was greatest where there were administrative interns.

**Conclusions:**

Placing interns in facilities to support non‐clinical tasks may improve HIV service delivery by contributing to improved HIV testing, treatment initiation and retention in care. Using youth interns as lay health workers may be an impactful strategy to strengthen the HIV response while supporting youth employment.

## 1 INTRODUCTION

While global resources allocated to HIV care and prevention have greatly expanded, efforts failed to reach 90‐90‐90 testing and treatment targets UNAIDS set for 2020 [1, 2]. South Africa, home to one‐in‐five people living with HIV globally [3], made significant progress towards these targets but must expand efforts to reach the more ambitious 95‐95‐95 targets set for 2030 [4, 5].

The shortage of workers at health facilities has long been a significant challenge for the delivery of HIV services in South Africa, as it is across sub‐Saharan Africa [6, 7]. The use of lay health workers (LHWs), such as lay counsellors, can bridge the staffing gap for HIV service delivery and has been promoted as a key strategy for achieving public health goals [7, 8]. Nonetheless, the uptake of LHWs has been slow in many places [9, 10, 11]. South Africa has relied heavily on LHWs to support HIV programmes, but the supply of LHWs is insufficient to meet current needs [12, 13].

While LHWs are scarce and health systems are understaffed, countries across sub‐Saharan Africa are experiencing a youth unemployment crisis [14, 15]. National 2019 unemployment statistics showed that, among people with secondary education, more than half in the 15–24 age group and one‐third in the 25–34 age group were unemployed in South Africa [16]. During the COVID pandemic, these numbers likely grew [17].

Youth Health Africa (YHA) was launched in 2018 in South Africa to address this dual burden of HIV‐related healthcare worker shortages and high youth unemployment [18]. YHA places young adults (18–34 years old) with a secondary education but no work experience as non‐clinical interns in health facilities, where they work as temporary LHWs. While LHW programmes are highly variable, many—but not all—programmes in sub‐Saharan Africa have improved patient care and health service delivery [19, 20, 21, 22, 23, 24, 25, 26, 27, 28, 29]. However, YHA is unique as it uses younger workers with less upfront training and shorter contracts than traditional LHW programmes. An assessment of the potential impact of these untapped human resources on the facility‐based HIV response in South Africa is needed.

## 2 METHODS

### 2.1 Study design

We conducted interrupted time series analyses using monthly programme data to explore the facility‐level impact of YHA on HIV service delivery and uptake. We examined differences in time trends for seven routinely collected HIV indicators related to HIV testing, treatment initiation and retention in care before and after the implementation of YHA, assuming the time trend observed prior to YHA would remain unchanged in the absence of YHA. The expected time trend thus served as the counterfactual, and changes from pre‐intervention trends for these indicators could cautiously be attributed to YHA [30]. To strengthen this interpretation, we conducted one additional analysis with an indicator that we would not expect to change; we compared differences in trends for study outcomes with this unrelated indicator. We also stratified each analysis by intern roles, number of interns and geography.

### 2.2 Setting

This study was based among Aurum‐affiliated facilities in Ekurhuleni, Gauteng and all districts in North West (NW) province in South Africa. NW is predominantly rural, while Ekurhuleni is urban (33 vs. 1609 persons/km^2^, respectively) [31, 32]. There are more people living with HIV in Gauteng, but NW has a higher HIV prevalence [33].

### 2.3 Youth Health Africa

YHA is a South African non‐governmental organization funded by business donations to support a national social responsibility policy (Broad‐Based Black Economic Empowerment) [18,34]. The first interns were placed in NW and Gauteng in late 2018; interns have since been placed across South Africa. Interns receive 1‐year placements as programmatic or administrative interns. Programmatic interns serve as HIV testing counsellors, patient navigators or tracers; administrative interns serve as file clerks or data capturers. YHA provides an introductory, 3‐ to 5‐day training on broad workplace skills; hosting facilities then provide technical training as necessary. While interns primarily work in their assigned roles, they support other tasks as needed. Multiple interns may be placed at a facility. Interns are placed when funding becomes available, meaning start dates vary.

### 2.4 Eligibility criteria

This study included all facilities in NW and Gauteng where Aurum was PEPFAR implementing partner and YHA interns were first placed November 2018–October 2019 (i.e. the first year of the programme). Aurum‐affiliated facilities were selected because YHA was first implemented in these facilities. The study defined YHA as implemented at a health facility if there was at least one intern who worked there ≥90 days; a 90‐day cut‐off was selected to ensure that interns were at facilities long enough to make a difference. (While internships normally lasted 1 year, interns could resign or be reassigned.)

### 2.5 Outcomes

**Figure 1:**
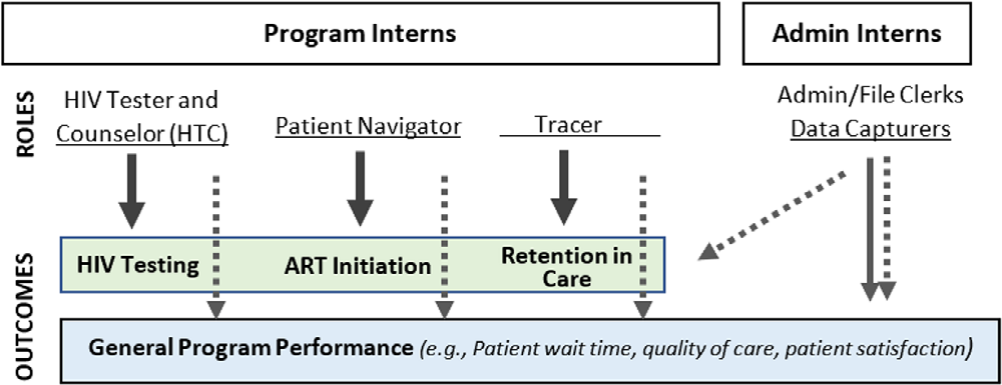
Theory of change for the Youth Heath Africa intern programme. Solid lines represent direct impact. Dotted lines represent indirect impact.

We analysed eight HIV service outcomes, selected based on YHA's theory of change (Figure 1) and the availability of data; we did not assess general programme performance. For testing, we analysed the number of people tested for HIV *(hts_tst)* and the number positive for HIV *(hts_pos)*. For treatment initiation, we analysed the number of people newly initiated on treatment (*tx_new)* and the number initiated on treatment within 14 days of diagnosis *(initiated_14days)*. For retention in care, we analysed the number of people with an outcome of loss to follow‐up (LTFU) *(outcome_ltf)*; LTFU was defined as a person missing more than 90 days despite three phone calls and a home visit to trace them. We also analysed the number of people completing viral load (VL) testing within 6 months of treatment initiation *(vl_completed_6mth)* and the number virally suppressed 6 months after treatment initiation *(vlsupp_6mth)*. The comparator outcome was the number of people initiated on isoniazid preventive therapy (IPT) *(ipt_start)*. We chose to analyse numbers rather than proportions due to data availability, data quality and stakeholder interest.

### 2.6 Defining intervention start

Start of the intervention was defined as the month a facility hosted its first intern; this varied by facility, occurring between November 2018 and October 2019. Due to non‐uniform start dates, we centred analyses on “time zero” (the month YHA started at a facility), not calendar time; we rounded this to the nearest month (e.g. if an intern started on 20 March, we rounded their start date up to April). Negative time corresponded to months before the facility had an intern; positive time corresponded to months since the first intern started at the facility.

### 2.7 Time period

This study includes 30 months of data from October 2017 to March 2020 for all outcomes except VL indicators, for which data were only available from October 2018 to March 2020. Data from months prior to the intervention start data were used to establish the time trend expected after intern placement if interns had no effect. This analysis did not extend past March 2020 due to programme disruptions associated with COVID‐19.

### 2.8 Data sources and preparation

Two datasets were used in this analysis: an intervention dataset to measure the exposure (intern placement) and a facility dataset to measure the outcome (HIV indicators). We created the intervention dataset by compiling YHA programme data on interns: health facilities where interns were placed, internship start and end dates, and intern roles. We compiled the outcome dataset using facility‐level data from TIER.Net, the national HIV surveillance system, as reported to Aurum. The outcome dataset included monthly data reported from October 2017 to March 2020 for all Aurum‐affiliated facilities in Gauteng and NW. The intervention dataset was aggregated by the facility and merged with the facility dataset in R 3.6.1 (R Core Team 2019, Vienna, Austria) by facility name. Data were cleaned in Stata 15 (StataCorp 2017, College Station, TX: StatCorp LLC).

As facilities had different start dates, facilities had variable numbers of data points in the pre‐intervention and intervention periods, with sparse data at extreme time points. Time points were considered extreme and excluded from the analysis if they included data from fewer than half of the facilities; this included time points ≥20 months before start of the intervention and those ≥11 months after start of the intervention. The final dataset contained 30 months of data, of which two‐thirds of time points (19/30 months) included data from all facilities.

### 2.9 Data analysis

We used segmented linear regression to model absolute numbers with restricted maximum likelihood estimates. We used “nlme” package in R to run analyses [35], adjusting for autocorrelation (AR1) and facility‐level clustering. We chose *a priori* to run models with slope changes but not immediate‐level changes because we hypothesized that YHA would result in gradual change. We ran all models using random intercepts. We selected parsimonious models to maximize interpretability, even if a more complex model may have fit the data better.

We conducted eight primary time series centred at time zero: one analysis for each outcome of interest and one for the comparator. The base model was as follows:

$$Y_{ijt}=\beta_{0}+\beta_{1}{\mathrm{timecentered}}_{ijt}+\beta_{2}{\mathrm{timeafterint}}_{ijt}+E_{ij}$$

In these models, β_1_ equalled the pre‐intervention period slope, β_1_ + β_2_ equalled the intervention period slope and β_2_ equalled the difference in slope between intervention and pre‐intervention periods. All time was measured in the number of months since the start of the intervention. We tested the null hypothesis that there was no difference in slope after the intervention (β_2_ = 0), which we assessed at the 0.05 significance level.

In addition to the primary analyses, we ran secondary analyses: three additional time series per outcome, stratified by region, number of interns and intern role. The stratified model was as follows:

$$\begin{array}{ccc} Y_{ijt} & = & \beta_{0}+\beta_{1}{\mathrm{timecentered}}_{ijt}+\beta_{2}{\mathrm{timeafterint}}_{ijt}\\ & & +\;\beta_{3}{\mathrm{stratavariable}}_{ijt}+\beta_{4}\mathrm{timecentered}*{\mathrm{stratavariable}}_{ijt}\\ & & +\;\beta_{5}\mathrm{timeafterint}*{\mathrm{stratavariable}}_{ijt}+E_{ij} \end{array}$$

In the stratified model, β_2_ equalled the difference in slope between intervention and pre‐intervention periods for strata 1, while β_2_ + β_5_ equalled this difference for strata 2, and β_5_ was the difference in change in slope between strata (difference‐in‐difference). For these secondary analyses, we assessed whether there was a change in slope from the pre‐ to intervention period for each stratum (null hypotheses: β_2_ = 0, β_2_ + β_5_ = 0) and whether this change differed by strata (null hypothesis: β_5_ = 0); both were assessed at the 0.05 significance level.

In addition, we conducted two sensitivity analyses, rerunning the primary analyses with (1) an immediate‐level change and (2) both random intercepts and slopes.

All models were plotted using package “ggplot2” in R [36].

### 2.10 Ethics

This study was approved by the University of Witwatersrand (Johannesburg) and determined to be non‐human subject research by the University of Washington (Seattle). No consent was required, as this research used routinely collected, facility‐level data.

## 3 RESULTS

### 3.1 Participating facilities

This analysis included 207 facilities, predominantly clinics, with 604 interns (Table 1). Two‐thirds of facilities were in NW, but 60% of interns worked in Gauteng. The number of interns per facility varied widely (1–18), with Gauteng facilities generally having more interns than NW facilities. More interns served in administrative than programme roles; almost two‐thirds of NW facilities had only administrative interns.

**Table 1:** Characteristics of the Youth Health Africa facility‐based intern programme as implemented in Aurum‐affiliated sites in Gauteng and North West provinces, South Africa (November 2018–October 2019).

	Gauteng (*N* = 74 facilities)	North West (*N* = 133 facilities)	Total (*N* = 207 facilities)
*Facility type*			
Clinic	64 (86.5%)	103 (77.4%)	167 (80.7%)
Community health centre	4 (5.4%)	21 (15.8%)	25 (12.1%)
Hospital	4 (5.4%)	7 (5.3%)	11 (5.3%)
Unspecified	2 (2.7%)	2 (1.5%)	4 (1.9%)
*Average no. of interns per facility (SD)*			
All interns	4.9 (3.8)	1.8 (1.3)	2.9 (2.9)
Programme interns	2.7 (2.4)	0.4 (0.6)	1.3 (1.9)
*Total interns per facility (%)*			
1 or 2	21 (28.4%)	110 (82.7%)	131 (63.3%)
3 or 4	19 (25.7%)	18 (13.5%)	37 (17.9%)
5 or more	34 (45.9%)	5 (3.8%)	39 (18.8%)
*Total facilities with programme interns (%)*	60 (81.1%)	46 (34.5%)	106 (51.2%)
*Avg no. rounds of intern placements (SD)*	3.0 (1.6)	1.2 (0.5)	1.8 (1.4)
*Avg no. of interns placed in first round (SD)*	1.6 (1.1)	1.5 (0.9)	1.6 (1.0)
*Avg age of interns (SD)*	24.9 (2.8)	24.4 (2.7)	24.7 (2.8)
Total no. of interns^a^	364 (60.2%)	240 (39.7%)	604 (100%)

Abbreviation: SD, standard deviation.

^a^ Row % is provided.

### 3.2 Primary analysis

**Figure 2:**
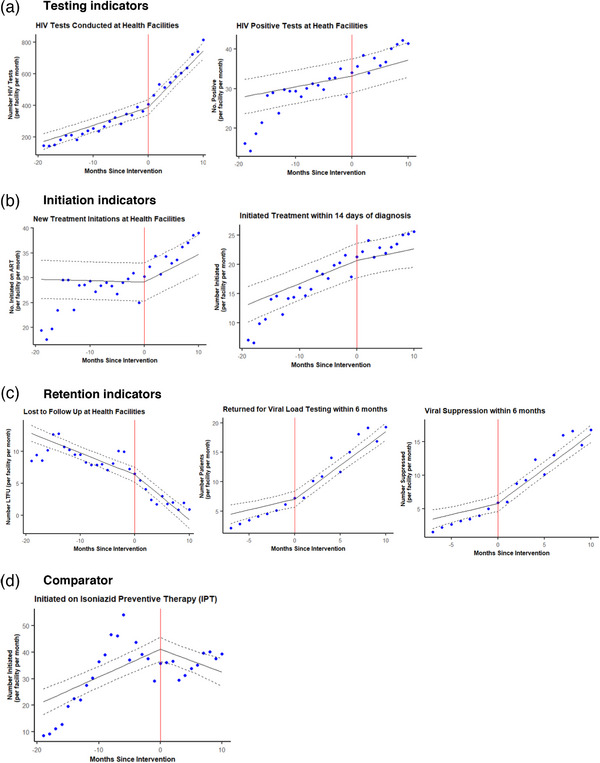
Primary time series analysis: Monthly reported HIV indicators from health facilities in Gauteng and North West, South Africa, centred at the time of Youth Health Africa intern placements at facility. Points are average outcomes per month. Black lines represent the model. Dotted lines represent corresponding 95% confidence intervals. Red lines indicate start of the intervention at each facility. Models cover calendar time October 2017–March 2020, except for viral load indicators which cover calendar time October 2018–March 2020.

After the introduction of YHA interns, there was a significant increase in trends for the number tested for HIV, initiated on treatment, completing VL testing and virally suppressed, and a significant decrease in trends for LTFU (Figure 2). These trends all moved in the direction of programme improvement. The change from pre‐intervention to intervention period was greatest for HIV testing, with a difference in slope of 24.5 (95% CI: 18.4, 30.5) people per month. The differences observed for other outcomes with significant findings were much smaller (<1 person per month) (Table 2). We did not observe significant changes in trends for the number positive for HIV or the number initiated on treatment within 14 days of diagnosis. The time trend for the comparator indicator showed no clear trends from pre‐intervention to intervention despite there being a significant decrease in slope.

**Table 2:** Results for the primary analysis regression models, including parameter coefficient and 95% confidence intervals.

	Intercept (β_0_)	Slope pre‐intervention (β_1_)	Slope post‐intervention (β1+β_2_)	Change in slope (β_2_)	*p*‐Value for β_2_
*Testing*					
Tested for HIV*	387 (339, 435)	11.3 (8.8, 13.9)	35.8 (27.2, 44.4)	24.5 (18.4, 30.5)	<0.0001
Positive for HIV	33.2 (28.9, 37.4)	0.27 (0.18, 0.36)	0.40 (0.07, 0.7 3)	0.13 (−0.11, 0.37)	0.2971
*Treatment initiation*					
Newly started on treatment*	29.1 (25.3, 33.0)	−0.03 (−0.11, 0.05)	0.56 (0.27, 0.85)	0.59 (0.37, 0.80)	<0.0001
Initiated on treatment within 14 days	20.7 (17.7, 23.6)	0.40 (0.28, 0.51)	0.20 (−0.21, 0.61)	−0.20 (−0.49, 0.10)	0.1944
*Retention in care*					
Lost to follow‐up*	6.4 (5.2, 7.6)	−0.34 (−0.41, −0.27)	−0.71 (−0.97, −0.44)	−0.37 (−0.56, −0.18)	0.0002
Viral load test completed*	7.1 (5.7, 8.4)	0.36 (0.14, 0.59)	1.15 (0.59, 1.70)	0.78 (0.45, 1.11)	<0.0001
Viral load suppressed*	5.8 (4.6, 7.0)	0.34 (0.13, 0.55)	1.04 (0.52, 1.55)	0.70 (0.39, 1.00)	<0.0001
*Comparator*					
Initiated IPT*	41.1 (36.6, 45.6)	1.04 (0.77, 1.31)	−0.87 (−1.86, 0.12)	−1.91 (−2.63, −1.19)	<0.0001

Note: Models are *Y = β_0_ + β_1_timecentered + β_2_timeafterint*, with change in slope (β_2_) being the parameter of interest. Analyses included 207 facilities.

^*^Statistically significant (*p*<0.05).

### 3.3 Secondary analyses

**Figure 3:**
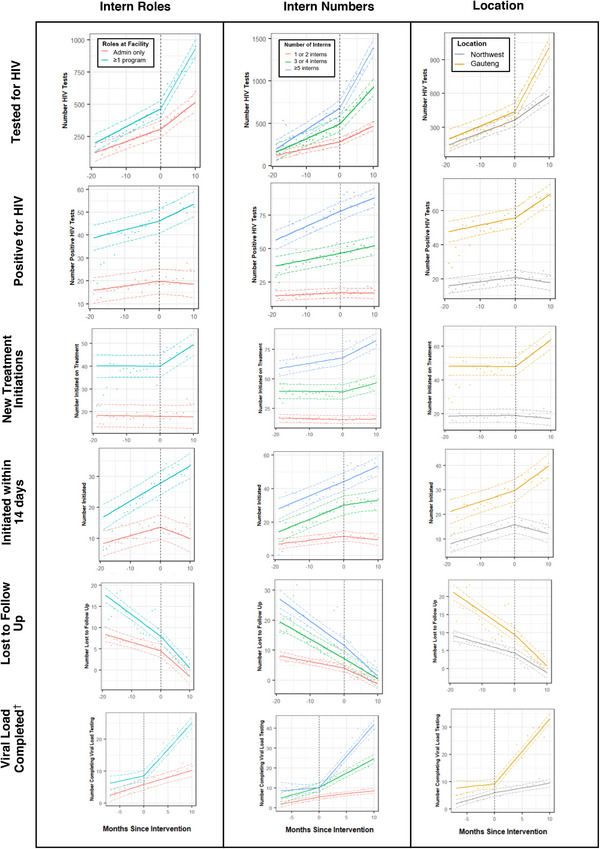
Secondary time series analyses (stratified): Monthly reported HIV indicators from health facilities in Gauteng and North West, South Africa, centred at the time of YHA intern placements at facility. Points are average outcomes per month. Solid lines represent the model. Dotted colour lines represent 95% confidence intervals. Dotted, vertical black line indicates the start of intervention at facility (time = 0 months). All models cover calendar time October 2017–March 2020, except for viral load indicators which cover calendar time October 2018–March 2020. †Viral load suppressed mirrored viral load completed.

Secondary analyses showed that for many outcomes, there were differences in level and trend between strata in the pre‐intervention period, which were often amplified in the intervention period (Figure 3). Pre‐intervention, facilities that received programme interns or ≥5 interns had a significantly stronger trend towards improvement for many outcomes than facilities that received only administrative interns or 1–2 interns. When YHA was implemented, facilities with programme interns, facilities with more interns and facilities in Gauteng experienced significant changes in trends for more outcomes than facilities with only administrative interns, facilities with 1–2 interns and facilities in NW (Table 3); the magnitude of changes from pre‐ to intervention period was generally greatest in these facilities (Figure 3). The LTFU analysis was unique as improvements in trends were found only for facilities with administrative interns, 1–2 interns and in NW. The analysis of treatment initiation within 14 days was also unique, as trends were significantly reduced after the intervention except in facilities with programme interns, ≥5 interns or in Gauteng. Full model results can be found in online Appendix A.

**Table 3:** Direction and significance of change in slope in the secondary analyses (intervention vs. pre‐intervention) (*p*<0.05).

*Change in slope*	Tested for HIV	HIV positive	New Txt initiations	Initiated within 14 days	Lost to follow‐up	Viral load completed	Viral load suppressed
*Intern type*							
Admin interns only	+			–	–		
Programme interns	+		+			+	+
Difference^a^ (programme vs. admin)	+	+	+	+		+	+
*Number of interns*							
1 or 2 interns	+			–	–		
3 or 4 interns	+		+				
5+ interns	+		+			+	+
Difference in trends (3–4 vs. 1–2)	+		+			+	+
Difference (5+ vs. 1–2)	+		+			+	+
*Region*							
North West	+	–		–	–		
Gauteng	+	+	+			+	+
Difference (Gauteng vs. North West)	+	+	+	+		+	+

Note: The “+” symbol indicates significant change in the positive direction; the “–” symbol indicates significant change in the negative direction; and blank cells indicate insignificant changes in slope.

^a^Difference‐in‐difference for the slope change observed for the two listed categories. Example: Change in slope observed in facilities with at least one programme intern minus change in slope observed in facilities with only admin interns.

### 3.4 Sensitivity analysis

Models run with a parameter for level change did not yield noticeable changes in effect size or statistical significance for difference in trends as compared to models used in the primary analysis, but analyses for HIV testing, HIV‐positive cases and new treatment initiation did have significant increases in level changes (online Appendix B). Models run with random intercepts and slopes did not converge for LTFU and IPT outcomes. Where models did converge, there were no changes in significance and minimal changes in effect size; VL indicators were the exception, which did not have a significant change in slope from pre‐ to post‐intervention in the new models (online Appendix C).

## 4 DISCUSSION

Our study suggests using youth interns as LHWs may be an impactful strategy to strengthen components of HIV service delivery at health facilities. Despite the young age and temporary nature of YHA LHWs, we observed significant improvements in trends for HIV testing, treatment initiations, LTFU, and VL testing and suppression after interns started at facilities; we did not observe changes for positive diagnoses or treatment initiation. Causal inference is strengthened as trends for the comparator did not follow a pattern based on YHA implementation, and changes observed generally aligned with YHA's theory of change; more change was observed with programme interns, who worked in roles that could directly impact HIV service indicators, and with more interns (i.e. a dose–response effect). Together, these findings signal that YHA, when implemented with sufficient interns, specifically programme interns, may help facilities strengthen aspects of HIV programming.

In many ways, our positive results are not surprising. Our finding that YHA is associated with an increase in HIV testing echoes past research that found HIV testing increased in facilities with lay counsellors, primarily because there were more people available to do HIV testing and counselling [19, 22, 23]. Similarly, our findings that treatment initiations increased with interns may be expected as prior research suggests peer navigators may improve social support and build trust among patients, which could lead to increased treatment uptake [24, 25, 27, 28], or that LHWs indirectly contribute to an increase in treatment initiations by improving clinic operations (e.g. wait times) [26]. This suggests the YHA approach may have positive impacts in line with traditional LHW programmes.

The analyses examining retention outcomes were more surprising. Reduction in LTFU was linked to administrative, not programme, interns. Administrative intern roles in data entry lead us to speculate that YHA may have affected LTFU by improving data quality. This would align with past research that suggests LTFU in South Africa is often a data quality problem [37, 38], but additional research is needed to ascertain if improvements in LTFU were due to improvements in data quality or true reductions. We are less confident in YHA's possible impact on VL indicators due to the lack of changes observed in the sensitivity analysis and less data being available for the analysis. VL indicators may have increased at all sites during the intervention period because 90‐90‐90 brought greater attention to VL monitoring—but these changes may have been accelerated by programme interns. Additional research is needed to elucidate how interns may have led to change in these retention outcomes.

It is also important to review outcomes where we observed no changes in trend after YHA implementation. Lack of change in positive HIV cases despite increased testing highlights that facility‐based testing, even if expanded, may struggle to find HIV‐positive individuals who do not know their status. The YHA approach could be more effective at finding new positives if, in addition to supporting HIV testing at facilities, interns facilitated alternative testing approaches in the community [39, 40]. Trends for early treatment initiation also failed to change after the intervention, even though overall treatment initiations increased. This may be because treatment initiation involves multiple steps, many of which cannot be directly affected by LHWs (e.g. initial clinical evaluations) [41]. Thus, interns might increase the number of people initiated on treatment, but not the speed at which each of these steps occurs.

Finally, we must consider how change varied by intern type, number and geography. Changes observed by intern type and number aligned with our theory of change; changes observed by location mirrored intern roles, likely due to most programme interns being in Gauteng. Interestingly, stratum‐specific differences observed in the pre‐intervention period suggest that pre‐existing differences between facilities may have influenced how many and what type of interns they received. Facilities that received more interns and programme interns may have been predisposed to success due to unmeasurable facility‐level factors (e.g. leadership, interest in programme improvement) that could have impacted HIV service outcomes both before and after intern arrival. This suggests that interns may have amplified improvements in these facilities, but other factors may also have contributed to positive change observed after their arrival.

### 4.1 Limitations

While a time series analysis is a robust quasi‐experimental method [30], our analysis was subject to limitations that temper causal inference. We could not account for underlying differences between facilities (e.g. staff‐to‐patient ratios or co‐existing programmes), which may have affected receipt and impact of YHA (i.e. unmeasured confounders). By centring this analysis at time zero, we helped mitigate confounding by temporal factors, but we recognize that health centres are complex entities with numerous quality improvement interventions ongoing at any time. Such interventions could have contributed to changes in trends observed in this analysis by affecting factors similar to those targeted by YHA (e.g. improved staff‐to‐patient ratios or clinic workflow). Moreover, we were unable to include control facilities in this analysis, as facilities participating in YHA were understood to differ from those not participating, which further tempers causal inference.

Our research was also limited by the use of programmatic data. The quality and breadth of information for exposure (intern placement) and outcomes (facility‐level HIV indicators) was limited by what was captured in routine practice. We also lacked data on YHA implementation (e.g. intern attendance) and facility operations (e.g. staff‐to‐patient ratios, co‐existing programmes), which could support a more nuanced analysis. In addition, our understanding of long‐term change was limited by the onset of COVID‐19, which restricted the length of time we could track after interns were placed at facilities. Finally, heterogeneity in outcomes between facilities resulted in an imperfect fit for some models.

### 4.2 Recommendations

While we cannot assert that YHA is responsible for all observed programme improvements, the results of our study do signal that YHA may help strengthen HIV service delivery, but more research is needed to understand this possible impact. A randomized trial could be useful to further examine the impact suggested by this study. Qualitative research would also be useful to understand other ways YHA could affect facilities, as LHW programmes may contribute to facility improvements not captured by health indicators, including general programme performance [20]. Qualitative research could also elucidate how this intervention may lead to change at facilities. Similarly, research is needed to understand how the young age of interns could have uniquely facilitated (or hindered) change.

Research on implementation outcomes is also necessary before recommending scale‐up [42]. We must understand the sustained impact of YHA, especially considering persistent, rapid intern turnover, which is fundamental to how the programme operates. Healthcare worker perceptions on YHA must also be elucidated, as programme acceptance and appropriateness are critical for success [42]. Finally, a costing study should be conducted to enable cost comparisons with traditional LHW programmes that are used to inform scale‐up.

Ideally, in the absence of trained healthcare workers, permanent LHWs can be used to fill human resource gaps and help reduce the healthcare worker shortage [7]. However, the ability of temporary, youth interns to serve as LHWs could be beneficial to health systems struggling to fund and fill LHW positions. YHA's focus on youth empowerment means it could tap into a large, underutilized labour supply (youth) and attract non‐traditional funders for public health projects, namely economic development agencies focused on youth (e.g. the African Development Bank) [43].

## 5 CONCLUSIONS

Our study suggests that the use of youth interns as LHWs may be an impactful strategy to strengthen HIV service delivery at health facilities. While interns did not appear to influence all aspects of HIV service delivery and may not affect outcomes if deployed in low numbers, their ability to positively impact any facility‐level outcomes suggests that youth could be an untapped source of LHWs to support health facilities deliver HIV programmes. As the HIV and unemployment epidemics in South Africa are mirrored across sub‐Saharan Africa, this innovative strategy may be of interest to other countries seeking to improve their HIV response while supporting youth employment. We recommend additional research to further assess impact and implementation outcomes prior to scale‐up.

## COMPETING INTERESTS

The Aurum Institute provides funding to Youth Health Africa. SR was an employee of Youth Health Africa. No other authors declare any potential competing interests.

## AUTHORS’ CONTRIBUTIONS

DT, SD, SC, GS and AD contributed to the conceptualization of this research. DT and AD acquired funding for this work. SC and SR contributed essential resources for this research, and DT curated the data for analysis. DT and SD selected the methods; DT performed the analysis with support and supervision from SD. DT visualized the results. SD and AD provided general supervision to this research. DT wrote the original draft of this manuscript; all authors reviewed and edited this manuscript.

## FUNDING

DT's time was supported by the National Centre for Advancing Translational Sciences of the National Institutes of Health under Award Number TL1 TR002318.

## DISCLAIMER

The content of this paper is solely the responsibility of the authors and does not necessarily represent the official views of the National Institutes of Health.

## Supporting information


**Appendix A**: Model parameters for secondary analysis.
**Appendix B**: Sensitivity Analysis (Immediate level change)
**Appendix C**: Sensitivity Analysis (Random slope and intercept)

## Data Availability

The data that support the findings of this study are available from the corresponding author upon reasonable request.
