# Supplementary material for: Does a youth intern programme strengthen HIV service delivery in South Africa? An interrupted time‐series analysis
Source: J Int AIDS Soc. 2023 Apr 12;26(4):e26083. doi: 10.1002/jia2.26083 (PMC10098286; doi:10.1002/jia2.26083)

## APPENDIX A: Model parameters for secondary analysis

**Table A. Model parameters representing slope change from each secondary analysis**

| **Outcome** | | **Point Estimate** | **95% CI Lower** | **95% CI Upper** |
| --- | --- | --- | --- | --- |
| **TESTED FOR HIV** | |  |  |  |
|  | **Region** |  |  |  |
| β2 | North West | 10.14894 | 2.948311 | 17.34956 |
| β2 + β5 | Gauteng | 44.41687 | 25.11324 | 63.7205 |
| β5 | Difference-in-difference | 34.26793 | 22.16492 | 46.37094 |
|  | **Number of Interns** |  |  |  |
| β2 | 1 or 2 interns | 10.28448 | 3.888887 | 16.68007 |
| β2 + β7 | 3 or 4 interns | 25.70552 | 5.964259 | 45.44679 |
| β2 + β8 | 5+ interns | 46.10401 | 26.0794 | 66.12863 |
| β7 | Difference-in-difference (3-4 vs. 1-2) | 15.42105 | 2.075372 | 28.76672 |
| β8 | Difference-in-difference (5+ vs 1-2) | 35.81954 | 22.19052 | 49.44856 |
|  | **Intern Type** |  |  |  |
| β2 | Admin interns only | 11.0579 | 2.532809 | 19.58299 |
| β2 + β5 | Program interns | 32.80174 | 12.55448 | 53.049 |
| β5 | Difference-in-difference | 21.74384 | 10.02167 | 33.46601 |
| **HIV POSITIVE** | |  |  |  |
|  | **Region** |  |  |  |
| β2 | North West | -0.5913 | -0.87624 | -0.30635 |
| β2 + β5 | Gauteng | 0.958115 | 0.192804 | 1.723426 |
| β5 | Difference-in-difference | 1.549412 | 1.069048 | 2.029775 |
|  | **Number of Interns** |  |  |  |
| β2 | 1 or 2 interns | -0.15653 | -0.44041 | 0.127348 |
| β2 + β7 | 3 or 4 interns | 0.065042 | -0.80882 | 0.9389 |
| β2 + β8 | 5+ interns | -0.14043 | -1.03271 | 0.751853 |
| β7 | Difference-in-difference (3-4 vs. 1-2) | 0.221572 | -0.36841 | 0.811552 |
| β8 | Difference-in-difference (5+ vs 1-2) | 0.016101 | -0.5923 | 0.624505 |
|  | **Intern Type** |  |  |  |
| β2 | Admin interns only | -0.34478 | -0.69496 | 0.005406 |
| β2 + β5 | Program interns | 0.358356 | -0.47134 | 1.188049 |
| β5 | Difference-in-difference | 0.703133 | 0.223623 | 1.182644 |

*Continued on next page*

| *Table A continued* | |  |  |  |
| --- | --- | --- | --- | --- |
| **Outcome** | | **Point Estimate** | **95% CI Lower** | **95% CI Upper** |
| **NEW TREATMENT INITIATIONS** | |  |  |  |
|  | **Region** |  |  |  |
| β2 | North West | -0.19128 | -0.43954 | 0.056991 |
| β2 + β5 | Gauteng | 1.595064 | 0.92827 | 2.261857 |
| β5 | Difference-in-difference | 1.786339 | 1.367811 | 2.204867 |
|  | **Number of Interns** |  |  |  |
| β2 | 1 or 2 interns | 0.114386 | -0.13645 | 0.365226 |
| β2 + β7 | 3 or 4 interns | 0.785695 | 0.013608 | 1.557782 |
| β2 + β8 | 5+ interns | 0.998427 | 0.209947 | 1.786906 |
| β7 | Difference-in-difference (3-4 vs. 1-2) | 0.671309 | 0.150063 | 1.192556 |
| β8 | Difference-in-difference (5+ vs 1-2) | 0.884041 | 0.346401 | 1.42168 |
|  | **Intern Type** |  |  |  |
| β2 | Admin interns only | 0.002807 | -0.30227 | 0.307879 |
| β2 + β5 | Program interns | 0.947904 | 0.225157 | 1.67065 |
| β5 | Difference-in-difference | 0.945097 | 0.527423 | 1.362771 |
| **NEW TREATMENT INITIATIONS WITHIN 14 DAYS** | | |  |  |
|  | **Region** |  |  |  |
| β2 | North West | -0.78067 | -1.13979 | -0.42155 |
| β2 + β5 | Gauteng | 0.560928 | -0.40317 | 1.525025 |
| β5 | Difference-in-difference | 1.341601 | 0.736624 | 1.946578 |
|  | **Number of Interns** |  |  |  |
| β2 | 1 or 2 interns | -0.45652 | -0.81117 | -0.10187 |
| β2 + β7 | 3 or 4 interns | -0.55221 | -1.64573 | 0.541322 |
| β2 + β8 | 5+ interns | 0.077579 | -1.03529 | 1.190446 |
| β7 | Difference-in-difference (3-4 vs. 1-2) | -0.09569 | -0.83456 | 0.64319 |
| β8 | Difference-in-difference (5+ vs 1-2) | 0.5341 | -0.22411 | 1.292315 |
|  | **Intern Type** |  |  |  |
| β2 | Admin interns only | -0.64673 | -1.06843 | -0.22502 |
| β2 + β5 | Program interns | -0.01724 | -1.01751 | 0.983034 |
| β5 | Difference-in-difference | 0.62949 | 0.050924 | 1.208056 |

*Continued on next page*

| *Table A continued* | |  |  |  |
| --- | --- | --- | --- | --- |
| **Outcome** | | **Point Estimate** | **95% CI Lower** | **95% CI Upper** |
| **LOST TO FOLLOW-UP** | |  |  |  |
|  | **Region** |  |  |  |
| β2 | North West | -0.29123 | -0.52496 | -0.0575 |
| β2 + β5 | Gauteng | -0.2543 | -0.88191 | 0.373313 |
| β5 | Difference-in-difference | 0.03693 | -0.35695 | 0.430813 |
|  | **Number of Interns** |  |  |  |
| β2 | 1 or 2 interns | -0.30975 | -0.5409 | -0.0786 |
| β2 + β7 | 3 or 4 interns | -0.02524 | -0.73818 | 0.687707 |
| β2 + β8 | 5+ interns | -0.21407 | -0.93967 | 0.511523 |
| β7 | Difference-in-difference (3-4 vs. 1-2) | 0.284512 | -0.19728 | 0.766306 |
| β8 | Difference-in-difference (5+ vs 1-2) | 0.095675 | -0.39877 | 0.590122 |
|  | **Intern Type** |  |  |  |
| β2 | Admin interns only | -0.40495 | -0.68061 | -0.12929 |
| β2 + β5 | Program interns | -0.22909 | -0.88303 | 0.424854 |
| β5 | Difference-in-difference | 0.17586 | -0.20243 | 0.554147 |
| **VIRAL LOAD TESTING COMPLETED (Within 6 months)** | | |  |  |
|  | **Region** |  |  |  |
| β2 | North West | -0.21978 | -0.55278 | 0.113225 |
| β2 + β5 | Gauteng | 2.148391 | 1.173526 | 3.123256 |
| β5 | Difference-in-difference | 2.368168 | 1.726306 | 3.01003 |
|  | **Number of Interns** |  |  |  |
| β2 | 1 or 2 interns | -0.20554 | -0.5108 | 0.09972 |
| β2 + β7 | 3 or 4 interns | 0.653459 | -0.34987 | 1.656792 |
| β2 + β8 | 5+ interns | 2.932201 | 1.745339 | 4.119063 |
| β7 | Difference-in-difference (3-4 vs. 1-2) | 0.858999 | 0.160926 | 1.557072 |
| β8 | Difference-in-difference (5+ vs 1-2) | 3.137741 | 2.25614 | 4.019343 |
|  | **Intern Type** |  |  |  |
| β2 | Admin interns only | -0.03895 | -0.4761 | 0.39819 |
| β2 + β5 | Program interns | 1.3369 | 0.267942 | 2.405857 |
| β5 | Difference-in-difference | 1.375852 | 0.744038 | 2.007667 |

| *Table A continued* | | |  |  |
| --- | --- | --- | --- | --- |
| **Outcome** | | | **Point Estimate** | **95% CI Lower** |
| **VIRAL LOAD SUPPRESSED (Within 6 months)** | | |  |  |
|  | **Region** |  |  |  |
| β2 | North West | -0.20207 | -0.5056 | 0.101459 |
| β2 + β5 | Gauteng | 1.894769 | 1.006277 | 2.783262 |
| β5 | Difference-in-difference | 2.096838 | 1.511874 | 2.681803 |
|  | **Number of Interns** |  |  |  |
| β2 | 1 or 2 interns | -0.19885 | -0.48116 | 0.083458 |
| β2 + β7 | 3 or 4 interns | 0.543131 | -0.38443 | 1.470693 |
| β2 + β8 | 5+ interns | 2.784358 | 1.688822 | 3.879894 |
| β7 | Difference-in-difference (3-4 vs. 1-2) | 0.741983 | 0.096732 | 1.387234 |
| β8 | Difference-in-difference (5+ vs 1-2) | 2.983211 | 2.169985 | 3.796436 |
|  | **Intern Type** |  |  |  |
| β2 | Admin interns only | -0.07415 | -0.47525 | 0.326944 |
| β2 + β5 | Program interns | 1.23856 | 0.257855 | 2.219265 |
| β5 | Difference-in-difference | 1.312712 | 0.733103 | 1.892322 |

## APPENDIX B: Sensitivity Analysis (Immediate level change)

**Figure B. Monthly reported HIV indicators from health facilities from model incorporating immediate level change (*Y = β_0_ + β_1_timecentered + β_2_int + β_3_timeafterint)*.** Models are centered at time of YHA intern placements at facility. Points are average outcomes per month. Black lines represent the model. Dotted lines represent the corresponding 95% confidence intervals. Red lines indicate the start of the intervention at each facility (time=0 months). All models cover calendar time October 2017-March 2020, except for viral load indicators which cover calendar time October 2018-March 2020.

***
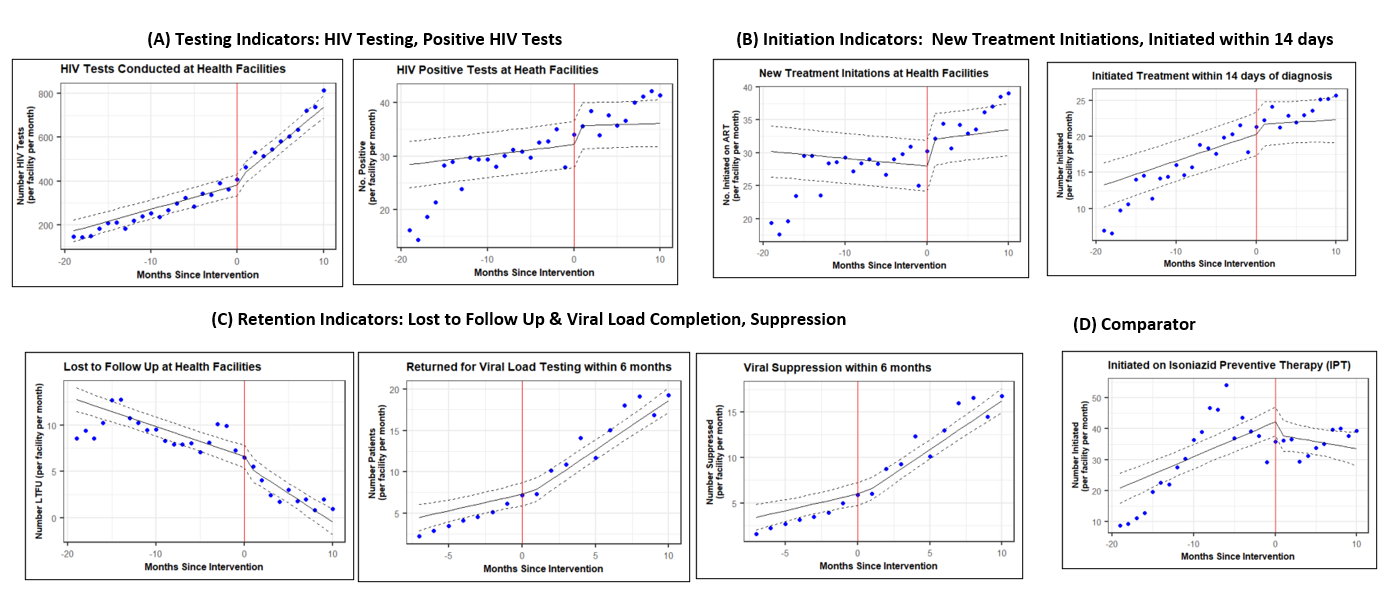
***

**Table B. Parameters for model with immediate level change
(*Y = β_0_ + β_1_timecentered + β_2_int + β_3_timeafterint)***

|  | **Point Estimate** | **95% CI Lower** | **95% CI Upper** | **Significance (p<0.05)** |
| --- | --- | --- | --- | --- |
| **HIV Testing** |  |  |  |  |
| β_0_ | 381.08765 | 332.7118 | 429.4635 | x |
| β_1_ | 10.95298 | 8.429353 | 13.47662 | x |
| β_2_ | 27.1325 | 5.080766 | 49.18424 | Sig |
| β_3_ | 22.1495 | 15.82013 | 28.47887 | Sig |
| **HIV Positive** |  |  |  |  |
| β_0_ | 32.1727944 | 27.87541 | 36.47018 | x |
| β_1_ | 0.1953552 | 0.099427 | 0.291283 | x |
| β_2_ | 3.4955249 | 1.855821 | 5.135229 | Sig |
| β_3_ | -0.1481338 | -0.42205 | 0.125784 | Not Sig |
| **Treatment New** |  |  |  |  |
| β_0_ | 28.0234459 | 24.16296 | 31.88393 | x |
| β_1_ | -0.1153454 | -0.19867 | -0.03202 | x |
| β_2_ | 3.8970664 | 2.439263 | 5.354869 | Sig |
| β_3_ | 0.2741153 | 0.035033 | 0.513197 | Sig |
| **Treatment within 14 days** |  |  |  |  |
| β_0_ | 20.3066287 | 17.31196 | 23.3013 | x |
| β_1_ | 0.3690165 | 0.251604 | 0.486429 | x |
| β_2_ | 1.3609958 | -0.12423 | 2.846222 | Not Sig |
| β_3_ | -0.3032958 | -0.62124 | 0.014651 | Not Sig |
| **LTFU** |  |  |  |  |
| β_0_ | 6.6213271 | 5.419002 | 7.823652 | x |
| β_1_ | -0.3223343 | -0.39772 | -0.24695 | x |
| β_2_ | -0.8592559 | -1.83239 | 0.11388 | Not Sig |
| β_3_ | -0.2985553 | -0.50343 | -0.09368 | Sig |
| **VL Completed** |  |  |  |  |
| β_0_ | 7.2758032 | 5.855592 | 8.696014 | x |
| β_1_ | 0.4013863 | 0.16567 | 0.637103 | x |
| β_2_ | -0.614975 | -1.56172 | 0.331769 | Not Sig |
| β_3_ | 0.7972245 | 0.464517 | 1.129932 | Sig |
| **VL Suppressed** |  |  |  |  |
| β_0_ | 5.9863602 | 4.724635 | 7.248085 | x |
| β_1_ | 0.3667501 | 0.150933 | 0.582568 | x |
| β_2_ | -0.4810965 | -1.34548 | 0.383286 | Not Sig |
| β_3_ | 0.7098319 | 0.404966 | 1.014698 | Sig |

## APPENDIX C: Sensitivity Analysis (Random slope and intercept)

**Figure C. Outcomes for base model using random slope and intercept (*Y = β_0_ + β_1_timecentered + β_2_timeafterint)***. Models for the outcomes “lost to follow-up” and “isoniazid preventive therapy” did not converge while using random slope and intercept. Models are centered at time of YHA intern placements at facility. Points are average outcomes per month. Black lines represent the model. Dotted lines represent the corresponding 95% confidence intervals. Red lines indicate the start of the intervention at each facility (time=0 months). All models cover calendar time October 2017-March 2020, except for viral load indicators which cover calendar time October 2018-March 2020.


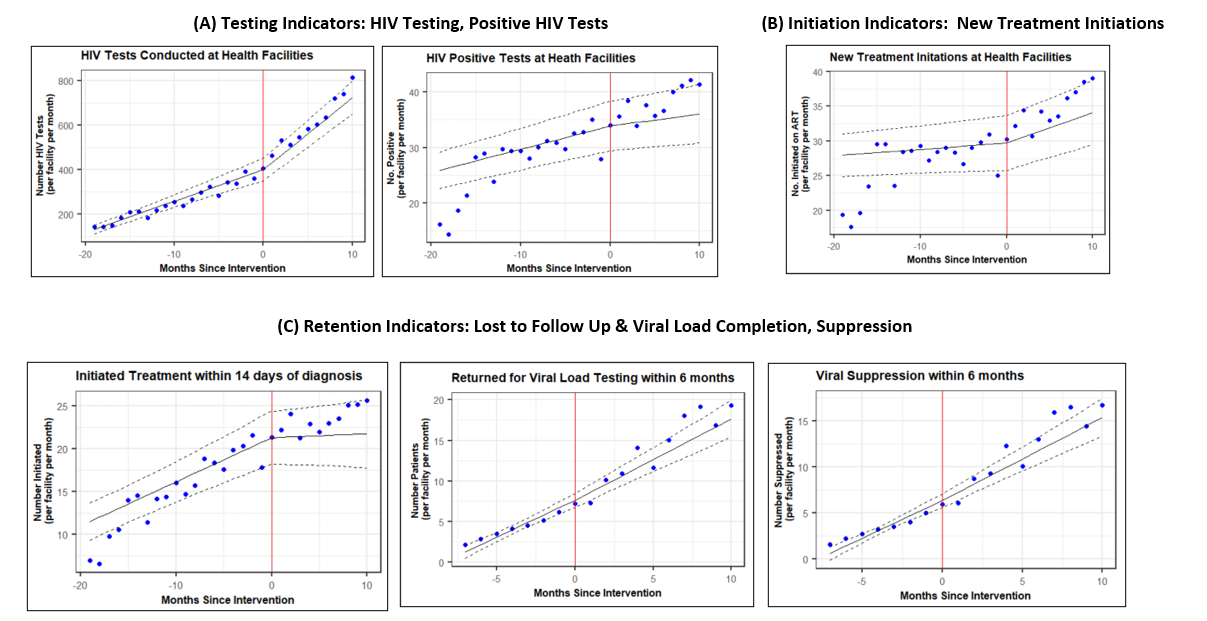

Supplement: Supplementary file 1 — Appendix A: Model parameters for secondary analysis. Appendix B: Sensitivity Analysis (Immediate level change) Appendix C: Sensitivity Analysis (Random slope and intercept) [file JIA2-26-e26083-s001.docx]
